# Enhancing alfalfa photosynthetic performance through arbuscular mycorrhizal fungi inoculation across varied phosphorus application levels

**DOI:** 10.3389/fpls.2023.1256084

**Published:** 2023-10-20

**Authors:** Dongjie Xia, Xiaoxia An, Ignacio F. López, Chunhui Ma, Qianbing Zhang

**Affiliations:** ^1^ College of Animal Science and Technology, Shihezi University, Shihezi, Xinjiang, China; ^2^ School of Agriculture and Environment, Massey University, Palmerston North, New Zealand

**Keywords:** alfalfa, AMF, phosphorus, net photosynthetic rate, dry matter yield

## Abstract

This study evaluated the effects of arbuscular mycorrhizal fungi inoculation on the growth and photosynthetic performance of alfalfa under different phosphorus application levels. This experiment adopts two-factors completely random design, and sets four levels of fungi application: single inoculation with *Funneliformis mosseae* (Fm, T_1)_, single inoculation with *Glomus etunicatum* (Ge, T_2_) and mixed inoculation with *Funneliformis mosseae* × *Glomus etunicatum* (Fm×Ge, T_3_) and treatment uninfected fungus (CK, T_0_). Four phosphorus application levels were set under the fungi application level: P_2_O_5_ 0 (P_0_), 50 (P_1_), 100 (P_2_) and 150 (P_3_) mg·kg^-1^. There were 16 treatments for fungus phosphorus interaction. The strain was placed 5 cm below the surface of the flowerpot soil, and the phosphate fertilizer was dissolved in water and applied at one time. The results showed that the intercellular CO_2_ concentration (C_i_) of alfalfa decreased at first and then increased with the increase of phosphorus application, except for light use efficiency (LUE) and leaf instantaneous water use efficiency (WUE), other indicators showed the opposite trend. The effect of mixed inoculation (T_3_) was significantly better than that of non-inoculation (T_0_) (*p* < 0.05). Pearson correlation analysis showed that C_i_ was significantly negatively correlated with alfalfa leaf transpiration rate (T_r_) and WUE (*p* < 0.05), and was extremely significantly negatively correlated with other indicators (*p* < 0.01). The other indexes were positively correlated (*p* < 0.05). This may be mainly because the factors affecting plant photosynthesis are non-stomatal factors. Through the comprehensive analysis of membership function, the indexes of alfalfa under different treatments were comprehensively ranked, and the top three were: T_3_P_2_>T_3_P_1_>T_1_P_2_. Therefore, when the phosphorus treatment was 100 mg·kg^-1^, the mixed inoculation of *Funneliformis mosseae* and *Glomus etunicatum* had the best effect, which was conducive to improving the photosynthetic efficiency of alfalfa, increasing the dry matter yield, and improving the economic benefits of local alfalfa in Xinjiang. In future studies, the anatomical structure and photosynthetic performance of alfalfa leaves and stems should be combined to clarify the synergistic mechanism of the anatomical structure and photosynthetic performance of alfalfa.

## 1 Introduction

Alfalfa (*Medicago sativa* L.) is a perennial legume herb with highly digestible fiber and high protein content, which can be used as hay and silage, making it an indispensable cultivated forage in arid and semi-arid areas of western China (Liu et al., 2021). Phosphorus (P) is an indispensable nutrient element in the process of plant growth and development, such as participating in the conversion and metabolism of chloroplast energy and the transport of leaf photosynthetic products in plant photosynthesis (Nasar et al., 2021). However, phosphorus anions are often adsorbed and precipitated in soil with other cations, resulting in limited availability of phosphorus in plants (Gao et al., 2016; Ning et al., 2019). Studies have shown that phosphorus deficiency acts as a key limiting factor for plant growth, which in turn affects high crop yields (Zhao et al., 2022a). Studies have shown that high concentrations of biogas slurry can promote the mobilization of colloidal phosphorus in rice fields, thereby promoting the absorption of phosphorus by rice (Niyungeko et al., 2018). Therefore, improving the phosphorus use efficiency of crops is of great significance for maintaining sustainable agriculture and crop cultivation.

Arbuscular mycorrhizal fungi (AMF) as a fungus that can establish symbiotic relationships with 80%-90% of the roots of land plants, including angiosperms, gymnosperms, ferns, and bryophytes, it can promote plant growth and induce plant stress resistance. (Frosi et al., 2016; Alotaibi et al., 2021). AMF can form mycelium structure with plant roots, and this structure increases the surface area of plant roots, improves the nutritional status of plants symbiotic, improves its absorption of nitrogen (N), phosphorus and mineral nutrients in the soil and then changes the stress resistance of plants (Sun et al., 2020; Yang et al., 2022), promotes the absorption of water by channel proteins and further improves crop water use efficiency, mineral nutrient nutrition and biomass (Qin et al., 2022). Studies have shown that AMF activates insoluble phosphorus by secreting phosphatases into the soil, and absorbs available phosphorus in the soil through high-affinity phosphorus transporters (Liu et al., 2020). And in the case of CO_2_ enrichment, AMF colonization can promote the absorption of phosphorus by plants (AbdElgawad et al., 2022). Therefore, AMF plays an important role in plant uptake and utilization of insoluble phosphorus and other nutrients in soil.

AMF can secrete cytokinins to increase chlorophyll content in plant leaves, thereby improving the yield of mixed sheepgrass (*Leymus chinensis*) and increasing photosynthesis of alfalfa (Shan et al., 2020). Studies have shown that changes in plant chlorophyll content can cause changes in plant photosynthetic activity, which in turn significantly affects plant development, growth and productivity (Zhang et al., 2017b). An important basis for photosynthesis in plants is leaves, and the photosynthetic capacity of leaves and the transport of photosynthetic products of their transport tissues play an important role in crop biomass (Li et al., 2020). Studies have shown that AMF can reduce the toxicity of beryllium to plants and promote photosynthesis of plants (Sheteiwy et al., 2022). Phosphorus application can improve the net photosynthetic rate, leaf chlorophyll content and nitrogen fixation capacity of roots, thereby significantly improving the production performance and nutritional quality of crops (Qi et al., 2013). Other studies have shown that phosphorolytic bacteria can improve plant leaf fence organization and leaf photosynthesis and increase their biomass (Sun et al., 2022). Therefore, fungus and phosphorus application play an important role in promoting the growth of alfalfa and improving photosynthesis.

At present, many studies have focused on inoculating a single AMF plant on alfalfa to improve dry matter yield and photosynthesis (Shan et al., 2020; Zhao et al., 2022a), however, the study on the relationship between photosynthesis and phosphorus interaction of alfalfa by simultaneous inoculation of two fungi under different phosphorus conditions is still missing. Therefore, in this study, the effects of inoculation of two fungi, *Funneliformis mosseae* (Fm) and *Glomus etunicatum* (Ge), on the dry matter yield, photosynthetic gas exchange parameters of alfalfa leaves and phosphorus utilization efficiency of alfalfa under different phosphorus application levels, were studied, in order to provide a theoretical basis for improving the measures for high-quality and high-yield alfalfa and the improvement of scientific and rational fertilization system. We hypothesized that: 1)when the phosphorus treatment was 100 mg·kg^-1^, the mixed inoculation of *Funneliformis mosseae* and *Glomus etunicatum* can significantly improve the photosynthetic efficiency and phosphorus utilization efficiency of alfalfa leaves; 2) the effect of mixed inoculation was significantly better than that of non-inoculation; 3) the top three treatments in terms of comprehensive ranking were T_3_P_2_, T_3_P_1_, and T_1_P_2_.

## 2 Materials and methods

### 2.1 Experimental materials

AMF uses *Funneliformis mosseae* (Fm) (BGC HK01) and *Glomus etunicatum* (Ge) (BGC GZ03C), the inoculant is a mixture of alfalfa plant roots, mycorrhizal fungal spores and extrarhizosphere mycelium, fungal spore density of 20~30 piece·g^-1^, purchased from Qingdao Agricultural Mycorrhizal Research Institute, China. AMF can form a good symbiotic relationship with the roots of most terrestrial plants, construct a root network in the soil, promote the absorption and utilization of nutrients by host plants, and thus plant growth can be promoted. Due to the drought and high light intensity in Xinjiang, WL366HQ was selected as the test plant for alfalfa varieties. The variety has a fall dormancy level of 5, high yield and cold tolerance. It can better adapt to the local environment in Xinjiang and is widely planted in Xinjiang.

Because the climatic conditions in Xinjiang are dry, the precipitation is scarce, and the soil is mostly gray desert soil, the soil used for potting is gray desert soil, which is collected from the 2nd Experimental Station of Shihezi University (44°18′N, 86°03′E), and the soil is naturally dried and sieved through 0.5 cm to remove stones and plant roots from the soil. The physical and chemical properties of the soil are shown in **Table 1**, the soil is sterilized at 121°C high temperature and humid heat for 2 h, and then naturally cooled for backup, and the potting substrate is sterilized soil and perlite 3:1 (V:V) mixed to prevent soil compaction.

**Table 1:** Basic physical and chemical properties of test soil.

Bulk density (g·cm^-3^)	Alkaline nitrogen (mg·kg^-1^)	Organic matter (g·kg^-1^)	Available phosphorus (mg·kg^-1^)	Total phosphorus (g·kg^-1^)	Available potassium (mg·kg^-1^)
1.47	71.8	24.1	18.3	0.23	135.5

### 2.2 Experimental design

The experiment was designed with two factors of complete randomization, with two factors: fungi application and phosphorus application. Fungi application was set at 4 levels: 10 g of *Funneliformis mosseae* (Fm, T_1_), 10 g of *Glomus etunicatum* (Ge, T_2_), 5 g of *Funneliformis mosseae* and 5 g of *Glomus etunicatum* (Fm×Ge, T_3_), with no fungi as the control (T_0_). Four more phosphorus levels were set under each application condition: P_2_O_5_ 0 (P_0_), 50 (P_1_), 100 (P_2_) and 150 (P_3_) mg·kg^-1^, and a total of 16 treatments were performed with 10 replicates of each treatment, for a total of 160 pots, and the pots were randomly placed.

The potting experiment was carried out in 2021-2022 in the experimental park of the College of Agriculture, Shihezi University (44°18′N, 86°03′E), and the experiment used pots with a size of 23 cm×17 cm×15 cm (pot mouth diameter× pot floor diameter × pot height), each pot containing about 3 kg of soil substrate, and the pots were disinfected with 75% alcohol before sowing. High-quality alfalfa seeds of uniform size and full grains were selected, sterilized with 10% H_2_O_2_ for 10 min, and then rinsed repeatedly with distilled water, and sown on May 1, 2021, with 10 seeds evenly distributed per pot. After the alfalfa three-leaf stage, five robust and uniform alfalfa seedlings were retained. In order to simulate the natural growth conditions of alfalfa, pots were placed in sunny places, and phosphorus-free Hoagland ‘s nutrient solution and equal amount of water were applied weekly during alfalfa seedlings. The phosphate fertilizer used was monoammonium phosphate (containing P_2_O_5_ 52%, N 12.2%), with its main component being P_2_O_5_, which has good water solubility and can be better absorbed and utilized by alfalfa, because the phosphate fertilizer had a small amount of N, so a certain amount of urea (containing N 46%) was supplemented under different phosphorus gradients in this experiment to make the N content under different phosphorus gradients consistent to offset the influence of nitrogen content in phosphate fertilizer on the experiment results. The fertilizer is applied with water droplets using the drip irrigation method of fertilizer with water, and the specific fertilization time is June 23, August 1, 2021, and April 16 and June 7, 2022. Alfalfa is harvested at the beginning of flowering (10%), on July 14, August 23, 2021, May 15, and June 30, 2022. Due to the insufficient environmental conditions for the first measurement of photosynthetic gas exchange parameters, the indicators were measured in the second stubble.

### 2.3 Alfalfa dry matter yield

In pots, three pots of alfalfa plants with uniform growth in the second stubble of the two years were selected, and the aerial part of the alfalfa plant was cut 2 cm from the surface of the potted soil for weighing, and the fresh weight was recorded. The fresh sample of alfalfa was dried at 105°C for 30 min, and then dried to constant weight at 65°C, the moisture content was determined and the dry matter yield was converted, the calculation formula was as follows: Dry matter yield = plant fresh weight × (1-moisture content), where moisture content refers to the calculation based on plant fresh weight.

### 2.4 Alfalfa photosynthetic pigment content

Chlorophyll is responsible for capturing light energy and driving the photosynthetic process. It is the most important pigment in photosynthesis. Chlorophyll a and chlorophyll b in fresh alfalfa leaves were extracted by absolute ethanol method (Cui and Yang, 2011), and the absorbance values were determined by spectrophotometer, and the total chlorophyll a, chlorophyll b and chlorophyll content in alfalfa leaves were calculated.

### 2.5 Alfalfa photosynthetic gas exchange parameters

Under cloudless conditions with sufficient light, the photosynthetic gas exchange parameters of well-grown alfalfa leaves were randomly determined by measuring the first flowering stage (10%) of the second stubble of two-year potted alfalfa on August 20, 2021 and June 27, 2022 at 11:00~13:00 a.m. The healthy intact and fully expanded leaflets in the middle of the third triple compound leaf of alfalfa plants from top to bottom were selected, and the photosynthetically active radiation (PAR) was controlled at about 1500 μmol·m^-2^·s^-1^, and each treated sample was measured in 3 replicates for analysis and averaged. Specific photosynthetic gas exchange parameters include net photosynthetic rate (P_n_, which represents the rate of carbon dioxide absorption and assimilation during photosynthesis, μmol·m^-2^·s^-1^), stomatal conductance (G_s_, which is the degree of stomatal opening, mmol·m^-2^·s^-1^), transpiration rate (T_r_, which represents the amount of water transpiration per unit leaf area in a certain period of time, mmol·m^-2^·s^-1^), intercellular CO_2_ concentration (C_i_, which reflects the utilization efficiency of CO_2_ by plants, μmol·mol^-1^), light use efficiency (LUE, which is an index to measure the light use efficiency and production level of crops in a certain area, mmol·mol^-1^) and alfalfa leaf instantaneous water use efficiency (WUE, which reflects the water utilization capacity of plant leaves, μmol·mmol^-1^), in which the light energy use efficiency and alfalfa leaf instantaneous water use efficiency are calculated by formulas, the specific calculation formula is as follows: LUE=P_n_/PAR; WUE=P_n_/T_r_.

### 2.6 Statistical analysis

Microsoft Excel 2010 was used for data processing, DPS 7.05 software (Data Processing System, China) was used for statistical analysis of data, two-factor experiment in a completely randomized design was used to statistically analyze the interaction between fungus treatment (T), phosphorus treatment (P) and fungus phosphorus (T×P), multiple comparisons were made by Duncan’s new complex polarity difference method, and the mapping software used Origin 2021 (OriginLabOriginPro, USA).

Because the performance of each treatment is different in different indexes, it is not comprehensive to evaluate the optimal bacterial phosphorus treatment with any single index (Zhang et al., 2017a). The membership function analysis method is used to comprehensively evaluate the optimal treatment, and the specific formula is: UX(+) = (X_ij_-X_imin_)/(X_imax_-X_imin_); UX(-) =1-UX(+), where X_ij_ is the measurement value of the jth index in the ith treatment; X_imax_ and X_imin_ are the maximum and minimum values of the jth indicator in all treatments, respectively. UX(+) is the value of the positive indicator membership function, and UX(-) is the value of the negative indicator membership function. The range of membership function values is 0-1.

## 3 Results

### 3.1 Effects of different treatments on the dry matter yield of alfalfa

**Figure 1:**
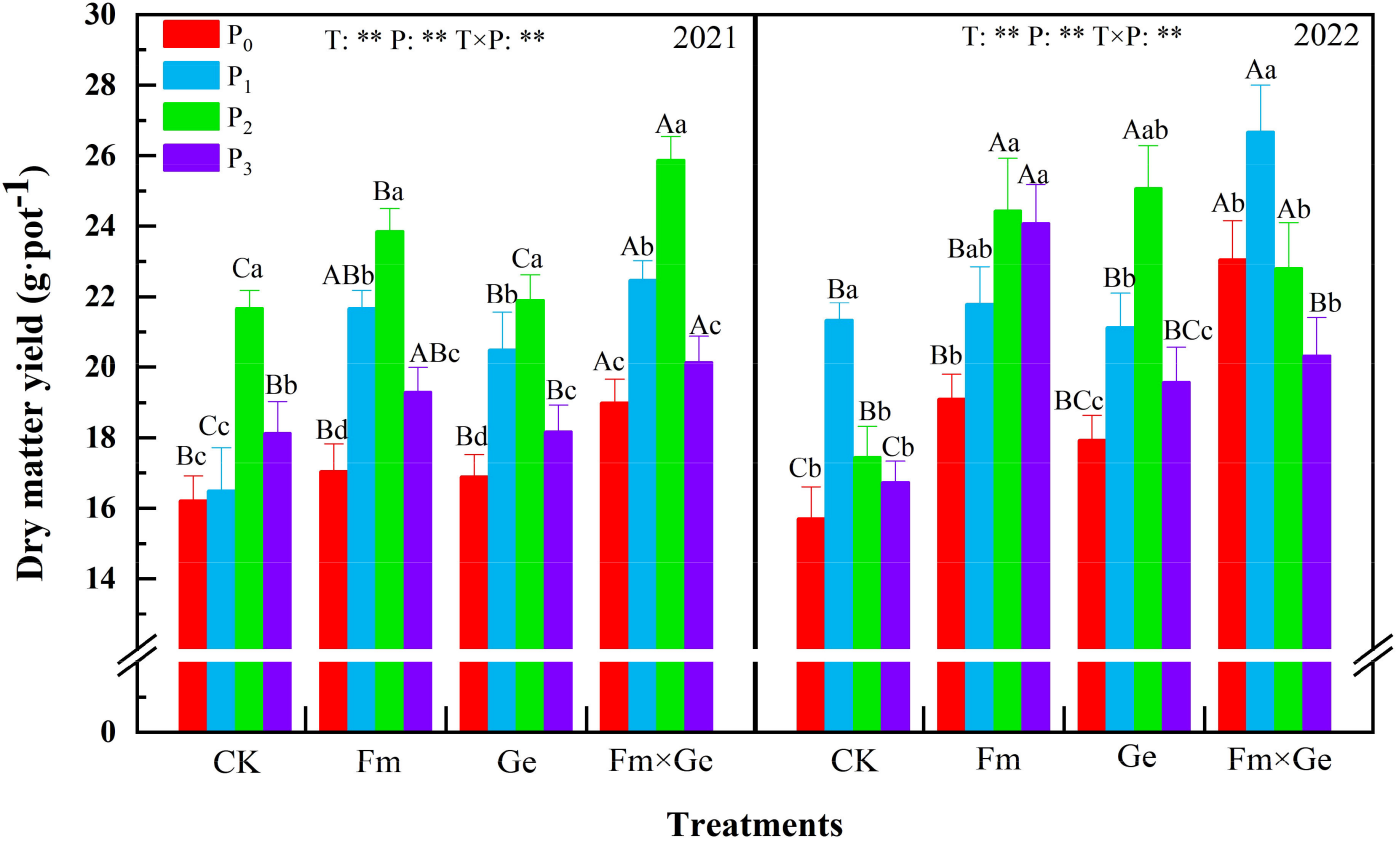
Dry matter yield of alfalfa under different treatments from 2021 to 2022. Note: T_0_, T_1_, T_2_, and T_3_ represent CK, *Funneliformis mosseae* (Fm), *Glomus etunicatum* (Ge), and *Funneliformis mosseae* × *Glomus etunicatum* (Fm×Ge), respectively. P_0_, P_1_, P_2_, and P_3_ represent 0 mg·kg^–1^, 50 mg·kg^–1^, 100 mg·kg^–1^, and 150 mg·kg^–1^, respectively. Different capital letters indicated significant difference in different fungus treatments under the same phosphorus application conditions (*p* < 0.05), differences small letters mean significant difference under the same fungus application conditions (*p* < 0.05). ** indicates significant difference extremely (*p* < 0.01). The value of n is 48.

According to the test results, dry matter yield of alfalfa exhibited an increase at lower P doses (P_0_-P_2_) and a decrease at higher doses (P_2_-P_3_) within the same fungal treatment condition (**Figure 1**). Except for P_1_ treatment significantly larger than P_0_, P_2_ and P_3_ in 2022 (*p* < 0.05), the dry matter yield of alfalfa treated with phosphorus treatment reached the highest value in P_2_ treatment, which was 25.87 g·pot^-1^ in 2021, which was 58.2% higher than that of T_0_P_0_ treatment. In 2022, it would be 26.68 g·pot^-1^, which is 59.35% higher than that of T_0_P_0_ treatment. The dry matter yield of alfalfa under P_2_ treatment exhibited a statistically significant increase compared to other treatments (*p* < 0.05).

When the phosphorus treatment was 100 mg·kg^-1^, the dry matter yield of double inoculated Fm and Ge alfalfa exhibited a statistically significant increase compared to no fungus treatment (*p* < 0.05), and the maximum value of T_3_ treatment was reached under P_2_ treatment. The effect of simultaneous inoculation of Fm and Ge was significantly better than that of single inoculation (*p* < 0.05). The dry matter yield of alfalfa under the treatment of fungus (T), phosphorus treatment (P) and fungus phosphorus interaction (T×P) in two years showed extremely significant differences (*p* < 0.01).

### 3.2 Effects of different treatments on photosynthetic pigment content of alfalfa

**Figure 2:**
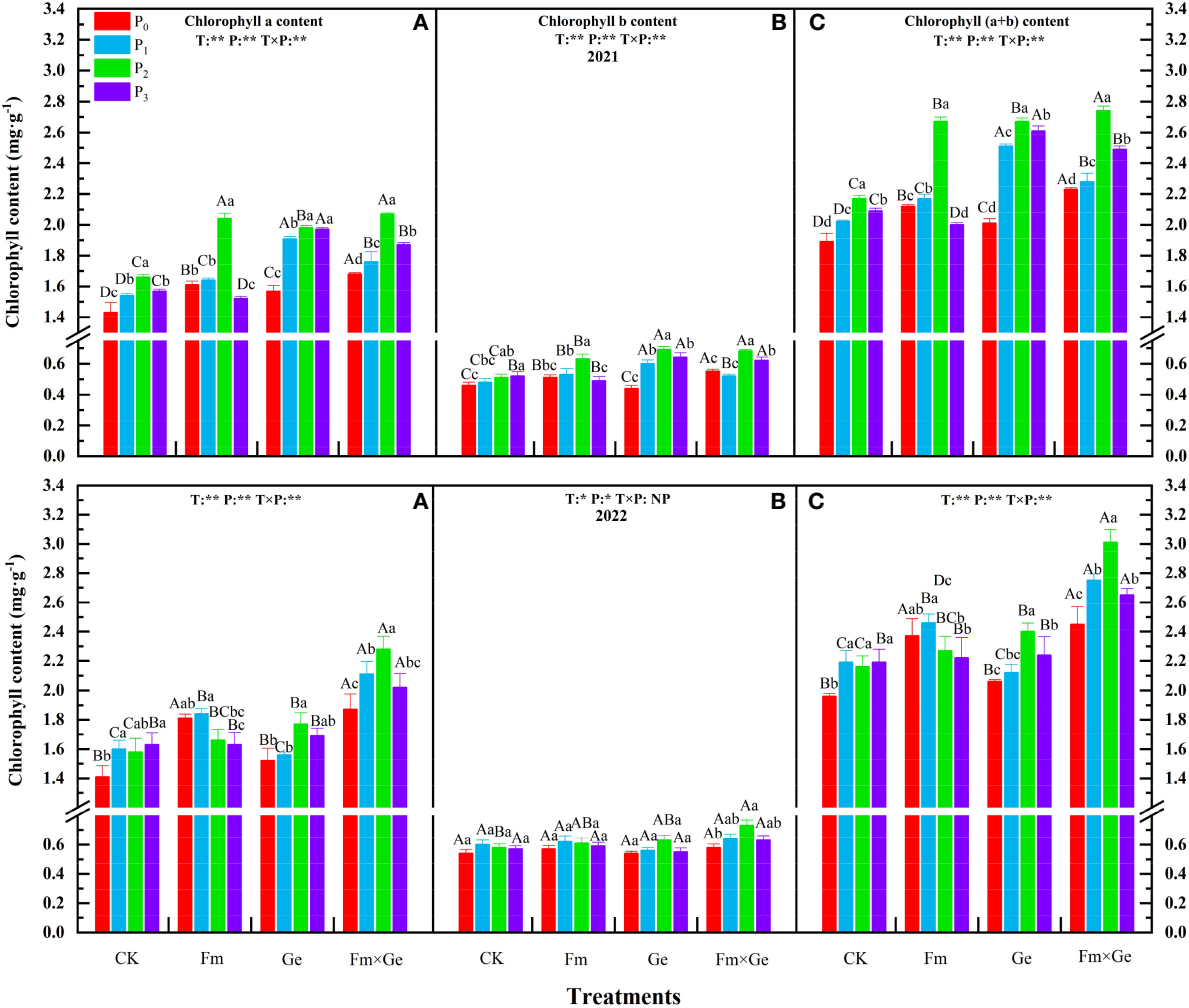
Chlorophyll content under different treatments from 2021-2022. **(A–C)** in the figure represent chlorophyll a content, chlorophyll b content, and total chlorophyll content, respectively. Different capital letters indicated significant difference in different fungus treatments under the same phosphorus application conditions (*p* < 0.05), differences small letters mean significant difference under the same fungus application conditions (*p* < 0.05). ** indicates significant difference extremely (*p* < 0.01). The value of n is 48.

There were obvious AMF × phosphorus interactions for chlorophyll a, chlorophyll b, and total chlorophyll contents. According to the test results, chlorophyll a, chlorophyll b, and total chlorophyll contents demonstrated an increase at lower P doses (P_0_-P_2_) and a decrease at higher doses (P_2_-P_3_) within the same fungal treatment condition (**Figure 2**). Except for the T_0_ treatment in 2021, the chlorophyll b content of alfalfa was significantly higher in the P_3_ treatment (*p* < 0.05), the chlorophyll a and total chlorophyll content was significantly higher in the P_3_ treatment (*p* < 0.05) under T_0_ condition in 2022, the chlorophyll b content was significantly higher in the P_1_ treatment (*p* < 0.05). The chlorophyll a, chlorophyll b and total chlorophyll contents of alfalfa was the highest when most of the phosphorus treatment was 100 mg·kg^-1^, and the P_2_ treatment exhibited a statistically significant increase compared to the P_0_ treatment (*p* < 0.05).

Under the condition of double inoculation of Fm and Ge fungal (T_3_), the chlorophyll a, chlorophyll b and total chlorophyll contents of alfalfa at the phosphorus treatment of 100 mg·kg^-1^ (P_2_) in 2021 and 2022 exhibited a statistically significant increase compared to the P_0_ treatment (*p* < 0.05). In 2021, the total chlorophyll content, chlorophyll a content and chlorophyll b content of alfalfa treated with T, P treatment and T×P showed extremely significant differences (*p* < 0.01), and in 2022, the chlorophyll a and total chlorophyll content of alfalfa between T, P treatment and T×P treatment showed extremely significant differences (*p* < 0.01). In 2022, the chlorophyll b content T and P treatment showed significant differences (*p* < 0.05).

### 3.3 Effects of different treatments on photosynthetic gas exchange parameters of alfalfa

**Figure 3:**
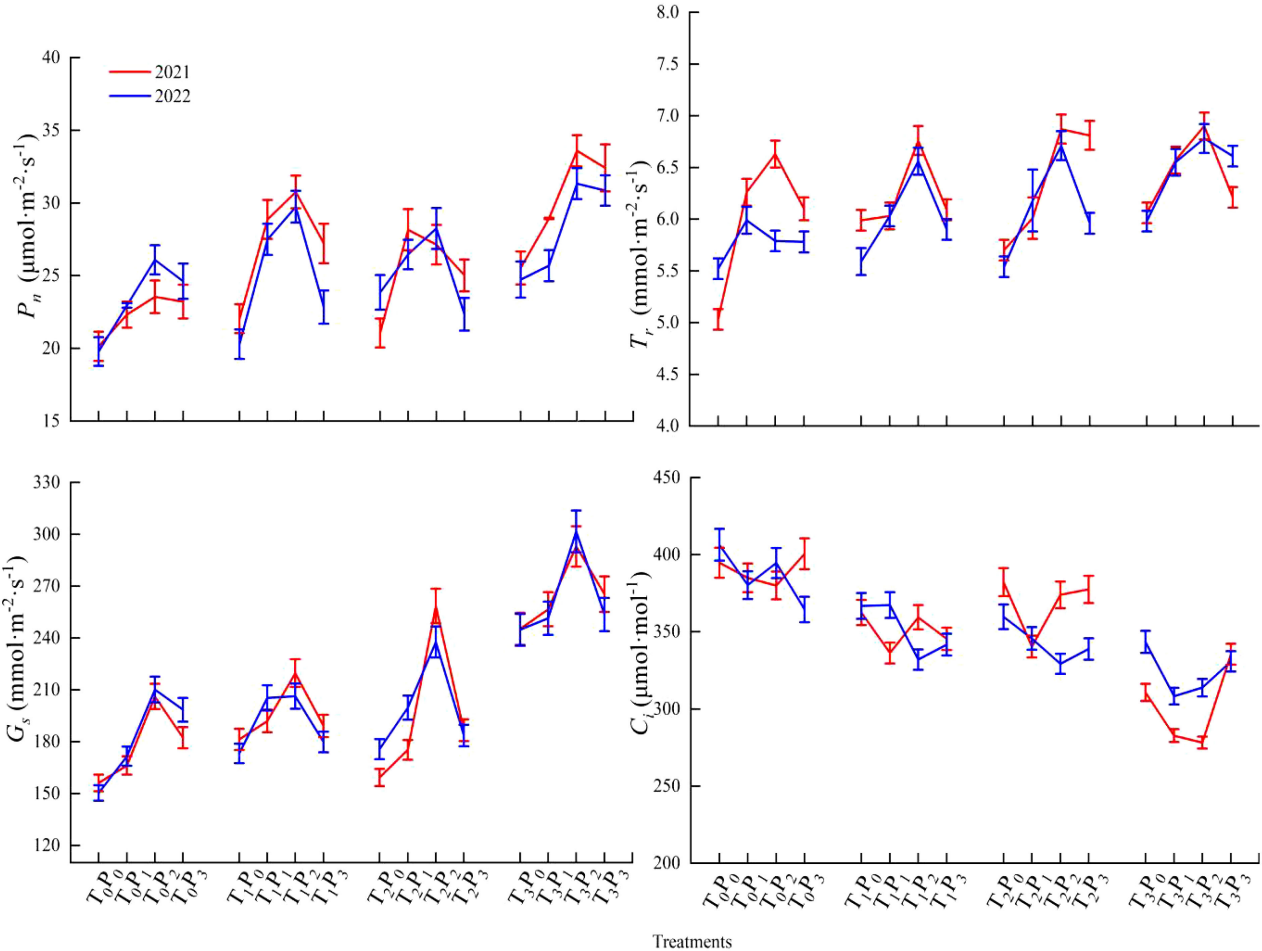
Photosynthetic gas exchange parameters of alfalfa under different treatments from 2021 to 2022.

Effects of two years of different phosphorus treatments on photosynthetic gas exchange parameters of alfalfa (**Figure 3**).

#### 3.3.1 Net photosynthetic rate

According to the test results, the net photosynthetic rate (P_n_) values of alfalfa exhibited an increase at lower P doses (P_0_-P_2_) and a decrease at higher doses (P_2_-P_3_) within the same fungal treatment condition. Except for 2021, the net photosynthetic rate demonstrated a statistically significant increase in the P_1_ treatment compared to P_0_, P_2_, and P_3_ under T_2_ treatment conditions (*p* < 0.05), the rest of the treatments demonstrated a statistically significant increase in the P_2_ treatment compared to P_0_, P_1_, and P_3_ (*p* < 0.05). Phosphorus treatment led to a significant increase in photosynthetic parameters compared to the non-phosphorus treatment (*p* < 0.05). Under the same phosphorus treatment, FmGe treatment > Fm/Ge treatment > CK treatment.

#### 3.3.2 Transpiration rate

According to the test results, the transpiration rate (T_r_) values of alfalfa exhibited an increase at lower P doses (P_0_-P_2_) and a decrease at higher doses (P_2_-P_3_) within the same fungal treatment condition. The transpiration rate values of alfalfa demonstrated a statistically significant increase in the P_2_ treatment compared to P_0_, P_1_, and P_3_ (*p* < 0.05). Phosphorus treatment led to a significant increase in photosynthetic parameters compared to the non-phosphorus treatment (*p* < 0.05). Under the same phosphorus treatment, FmGe treatment > Fm/Ge treatment > CK treatment.

#### 3.3.3 Stomatal conductance

According to the test results, the stomatal conductance (G_s_) values of alfalfa exhibited an increase at lower P doses (P_0_-P_2_) and a decrease at higher doses (P_2_-P_3_) within the same fungal treatment condition. The stomatal conductance demonstrated a statistically significant increase in the P_2_ treatment compared to P_0_, P_1_, and P_3_ (*p* < 0.05). Phosphorus treatment led to a significant increase in photosynthetic parameters compared to the non-phosphorus treatment (*p* < 0.05). Under the same phosphorus treatment, FmGe treatment > Fm/Ge treatment > CK treatment.

#### 3.3.4 Intercellular CO_2_ concentration

The intercellular CO_2_ concentration (C_i_) value of alfalfa exhibited an increase at lower P doses (P_0_-P_2_) and a decrease at higher doses (P_2_-P_3_) within the same fungal treatment condition. Except for 2021, the intercellular CO_2_ concentration of P_1_ exhibited a statistically significant decrease compared to other treatments under T_1_ and T_2_ treatment, P_3_ exhibited a statistically significant decrease compared to other treatments under T_0_ treatment in 2021 and P_1_ exhibited a statistically significant decrease compared to other treatments under T_3_ treatment in 2022, the rest was significantly lower in P_2_ treatment (*p* < 0.05). Under the same phosphorus treatment, T_0_ treatment exhibited a statistically significant increase compared to other treatments (*p* < 0.05).

### 3.4 Effects of different treatments on light energy use efficiency and instantaneous water use efficiency of alfalfa leaves

#### 3.4.1 Light energy use efficiency

**Figure 4:**
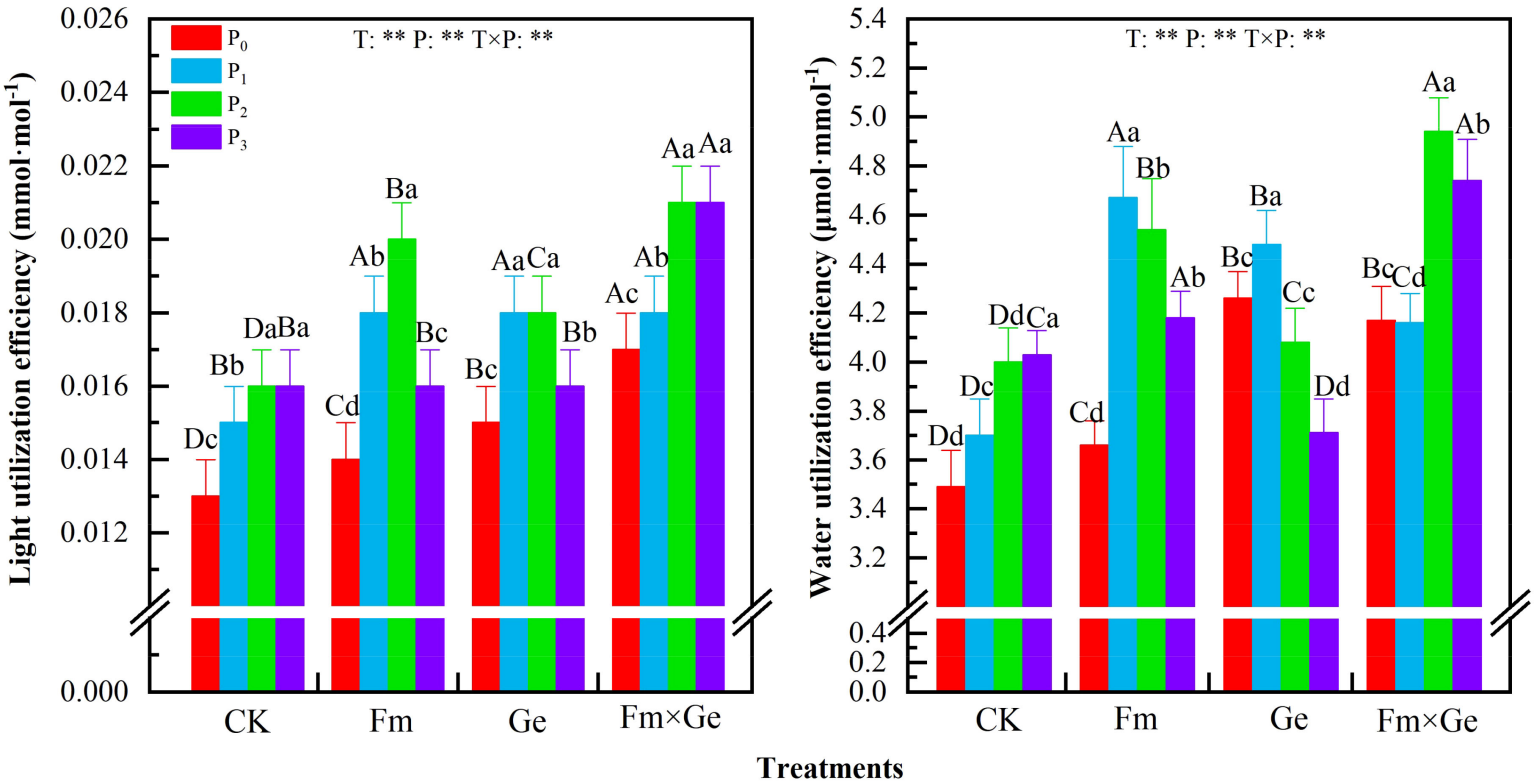
Light use efficiency and Water use efficiency of alfalfa leaves under different treatments from 2021-2022. Different capital letters indicated significant difference in different fungus treatments under the same phosphorus application conditions (*p* < 0.05), differences small letters mean significant difference under the same fungus application conditions (*p* < 0.05). ** indicates significant difference extremely (*p* < 0.01). The value of n is 48.

According to the test results, the light use efficiency of alfalfa in two years exhibited an increase at lower P doses (P_0_-P_2_) and a decrease at higher doses (P_2_-P_3_) within the same fungal treatment condition (**Figure 4**). The light use efficiency of alfalfa under P_2_ treatment exhibited a statistically significant increase compared to other treatments (*p* < 0.05).

When the phosphorus treatment was 100 mg·kg^-1^, the light use efficiency of alfalfa under T_3_ treatment exhibited a statistically significant increase compared to T_0_ treatments (*p* < 0.05). The effect of simultaneous inoculation of Fm and Ge was significantly better than that of single inoculation (*p* < 0.05). The light use efficiency of alfalfa under the fungus treatment (T), phosphorus treatment (P) and fungus phosphorus interaction (T×P) in two years showed extremely significant differences (*p* < 0.01).

#### 3.4.2 Instantaneous water use efficiency

According to the test results, the instantaneous water use efficiency of alfalfa in two years exhibited an increase at lower P doses (P_0_-P_1_) and a decrease at higher doses (P_1_-P_3_) within the same fungal treatment condition (**Figure 4**). Except that under T_0_ condition, the instantaneous water use efficiency of alfalfa leaves under P_3_ treatment exhibited a statistically significant increase compared to other treatments (*p* < 0.05), and under T_3_ condition, the instantaneous water use efficiency of alfalfa leaves under P_2_ treatment exhibited a statistically significant increase compared to other treatments (*p* < 0.05). The instantaneous water use efficiency of alfalfa was significantly higher in the P_1_ treatment (*p* < 0.05).

When the phosphorus treatment was 100 mg·kg^-1^, the instantaneous water use efficiency of alfalfa under T_3_ treatment exhibited a statistically significant increase compared to T_0_ treatment (*p* < 0.05). The effect of simultaneous inoculation of Fm and Ge was significantly better than that of single inoculation (*p* < 0.05). The instantaneous water use efficiency of leaves of alfalfa under the fungus treatment (T), phosphorus treatment (P) and fungus phosphorus interaction (T×P) in two years showed extremely significant differences (*p* < 0.01).

### 3.5 Correlation analysis of alfalfa indicators under different treatments

**Figure 5:**
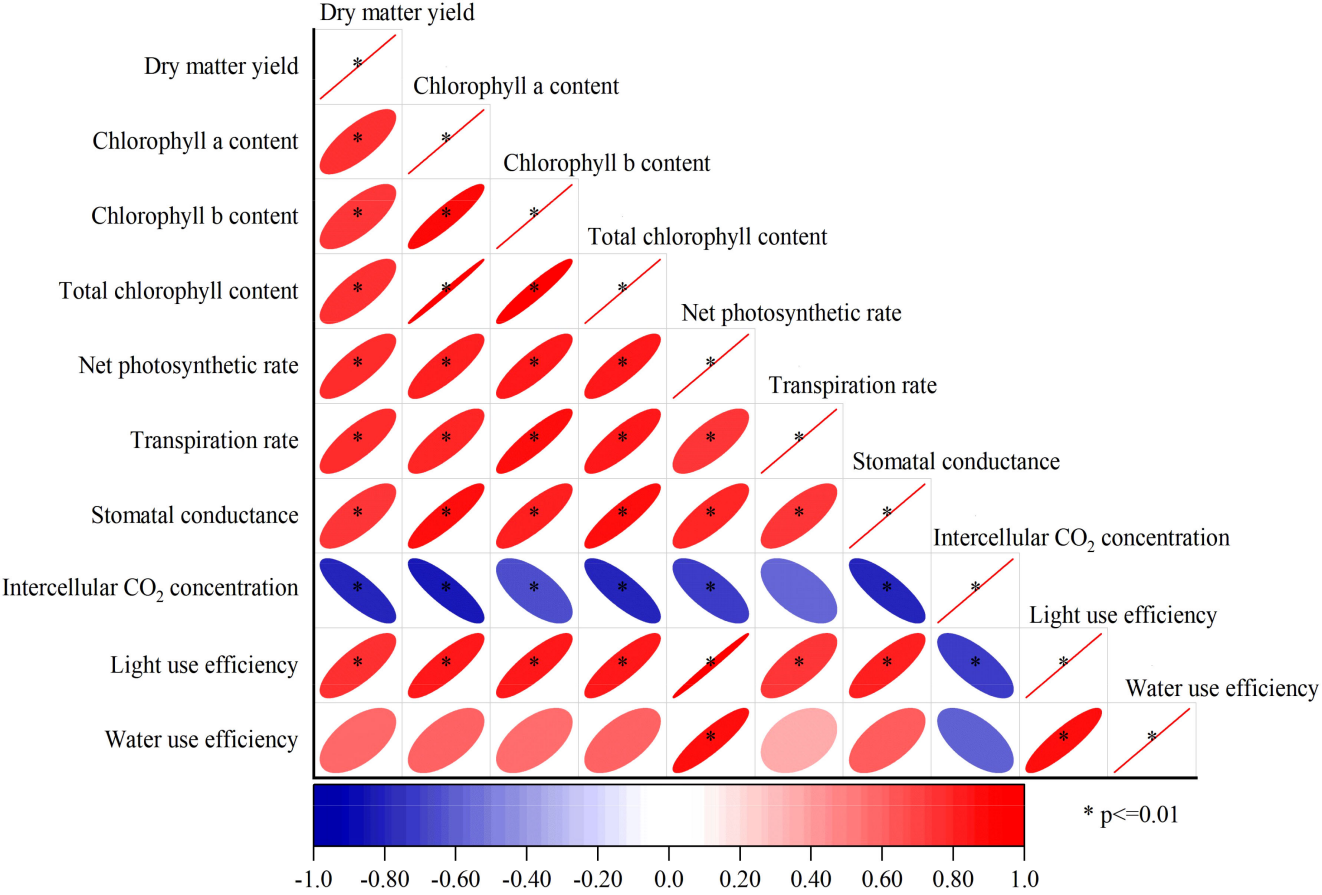
Correlation analysis of alfalfa indexes under different treatments. *Significant correlation was found at the 0.01 level (bilateral).

Pearson correlation analysis showed that except for the instantaneous water use efficiency of alfalfa leaves and intercellular CO_2_ concentration, the other indexes were extremely significantly positively correlated each other (*p* < 0.01) (**Figure 5**). Alfalfa leaves were extremely positively correlated with net photosynthetic rate and light use efficiency (*p* < 0.01), while alfalfa leaf water use efficiency was positively correlated with dry matter yield, chlorophyll a, chlorophyll b, total chlorophyll content and stomatal conductance (*p* < 0.05), but not significantly positively correlated with transpiration rate (*p* > 0.05). Intercellular CO_2_ concentration (C_i_) was extremely negatively correlated with dry matter yield, chlorophyll a content, chlorophyll b content, total chlorophyll content, leaf net photosynthetic rate, stomatal conductance and light use efficiency (*p* < 0.01), and intercellular CO_2_ concentration was negatively correlated with transpiration rate and instantaneous water use efficiency of alfalfa leaves (*p* < 0.05).

### 3.6 Comprehensive evaluation of membership functions

In order to avoid one-sidedness in the analysis of a single index and evaluate the optimal phosphorus and fungal application treatment (Sun et al., 2019), the membership function analysis evaluated eight key indicators of alfalfa performance (**Table 2**). The net photosynthetic rate, transpiration rate, stomatal conductance, light energy use efficiency and instantaneous water use efficiency of alfalfa leaves were all positive indicators, while the intercellular CO_2_ concentration was negative. The top three treatments in terms of comprehensive ranking were T_3_P_2_, T_3_P_1_, and T_1_P_2_.

**Table 2:** Comprehensive evaluation of alfalfa indexes.

Index	Dry matter yield	Total chlorophyll content	Net photosynthetic rate	Transpiration rate	Stomatal conductance	Intercellular CO_2_ concentration	Light use efficiency	Water use efficiency	Average	Rank
T_0_P_0_	0.000	0.000	0.000	0.290	0.000	1.000	0.000	0.000	0.161	16
T_0_P_1_	0.343	0.189	0.214	0.545	0.109	0.829	0.171	0.171	0.321	13
T_0_P_2_	0.418	0.253	0.389	0.599	0.381	0.874	0.127	0.127	0.396	12
T_0_P_3_	0.171	0.226	0.317	0.427	0.258	0.217	0.786	0.786	0.399	11
T_1_P_0_	0.246	0.337	0.096	0.331	0.167	0.658	0.343	0.343	0.315	14
T_1_P_1_	0.669	0.411	0.658	0.484	0.315	0.536	0.466	0.466	0.501	7
T_1_P_2_	0.950	0.575	0.823	0.885	0.415	0.478	0.528	0.524	0.652	3
T_1_P_3_	0.665	0.195	0.405	0.462	0.217	0.458	0.545	0.545	0.437	9
T_2_P_0_	0.168	0.116	0.199	0.000	0.099	0.718	0.283	0.283	0.233	15
T_2_P_1_	0.564	0.411	0.588	0.525	0.238	0.453	0.550	0.550	0.485	8
T_2_P_2_	0.874	0.642	0.618	0.968	0.658	0.530	0.468	0.468	0.650	5
T_2_P_3_	0.338	0.526	0.298	0.709	0.221	0.597	0.405	0.405	0.437	10
T_3_P_0_	0.588	0.437	0.413	0.478	0.637	0.300	0.703	0.703	0.532	6
T_3_P_1_	1.000	0.621	0.588	0.822	0.700	0.000	1.004	1.004	0.717	2
T_3_P_2_	0.972	1.000	1.000	1.000	1.000	0.004	1.000	1.000	0.872	1
T_3_P_3_	0.496	0.679	0.934	0.726	0.738	0.358	0.644	0.644	0.652	4

## 4 Discussion

### 4.1 Effect of AMF inoculation on the dry matter yield of alfalfa under different phosphorus application conditions

Alfalfa, as a perennial legume forage, has always been known for its high quality and high yield. Dry matter yield is one of the important representatives of alfalfa production performance index. The intricate relationship between phosphorus application and AMF inoculation significantly shapes alfalfa’s dry matter yield. Studies have shown that phosphorus application and AMF inoculation can significantly increase the phosphorus use efficiency and leaf chlorophyll content of alfalfa, thereby increasing the aboveground biomass of plants (Mickky et al., 2018). The results showed that, with the increase of phosphorus application, the dry matter yield of alfalfa increased at the lower dose of P (P_0_-P_2_), while it decreased at the higher dose of P (P_2_-P_3_), and the application of phosphorus fertilizer in alfalfa was better than that without phosphorus fertilizer (*p* < 0.05). When the amount of phosphorus (P_2_O_5_) reached 150 mg·kg^-1^, the dry matter yield of alfalfa decreased, which may be due to the high concentration of phosphorus, which caused a significant decrease in the diversity of AMF in the mycorrhizal community of alfalfa root and outside the root, and the colonization rate also decreased, which affected plant growth and reduced plant dry matter yield (Hinsinger, 2001). Therefore, the appropriate phosphorus application level is helpful to increase the yield.

In addition, high concentrations of phosphorus were found to damage the branches, vesicles and root fungal activity of maize (*Zea mays* L.) roots after AMF inoculation and phosphorus application (Chen et al., 2015.). In this study, under the condition of phosphorus application of 100 mg·kg^-1^, Fm × Ge treatment was significantly higher than that of the control group (*p* < 0.05). It can be seen that inoculation of the two fungi has a significant positive effect on the growth of alfalfa, the main reason may be that AMF inoculation can significantly reduce the content of phosphorus phytate in alfalfa plants, and the arbuscular mycorrhizal fungal mycelium can absorb phosphorus from the soil and transfer it to the roots of alfalfa (Sun et al., 2022). Studies have shown that inoculation of sweet potatoes (*Ipomoea batatas* (L.) *Lam*.) with arbuscular mycorrhizal fungi is able to exhibit higher levels of photosynthetic pigments, enhance chlorophyll fluorescence and net photosynthetic rate under dehydration stress (Yooyongwech et al., 2014), resulting in better growth and yield traits (Yooyongwech et al., 2016). Therefore, the increase in the dry matter yield of alfalfa is due to the accumulation of photosynthetic products and the increase of available phosphorus content in the soil.

### 4.2 Effect of AMF inoculation on photosynthetic characteristics of alfalfa under different phosphorus application conditions

Photosynthesis is the basis for the formation of organic matter, oxygen and dry matter yield in plants, and improving plant photosynthetic efficiency and chlorophyll content is of great significance for increasing the accumulation of plant dry matter yield and improving the maintenance of plant nutritional quality (Sun et al., 2022). Phosphorus has a very close relationship with the photosynthesis of plants. Studies have shown that ATP and enzymes in plant photosynthesis require the participation of phosphorus, and the increase of phosphorus in plants is beneficial to the progress of plant photosynthesis, and the assimilation of CO_2_ is directly related to the growth, development and photosynthesis of plants (Balestrini et al., 2020; Valkov et al., 2020). The results showed that, with the increase of phosphorus application, the net photosynthetic rate, transpiration rate and stomatal conductance of alfalfa increased at the lower dose of P (P_0_-P_2_), while it decreased at the higher dose of P (P_2_-P_3_), which may be because when the phosphorus content in the soil is insufficient for the phosphorus requirement of plants, the low phosphorus content will affect the opening and closing of the stomata in the leaves of plants, limit the regeneration of ribulose diphosphatase in alfalfa leaves (Zhang et al., 2016), and hinder the synthesis of ribulose diphosphate, which in turn affects the progress of alfalfa photosynthesis (Qi et al., 2013), photosynthesis is limited by the regeneration ability of RuBP in Calvin cycle. Rubisco is the rate-limiting enzyme of photosynthetic carbon assimilation, and its activity directly affects the photosynthetic rate of light saturation(Qi et al., 2013) and the application of phosphate fertilizer helps to improve the photosynthesis of alfalfa. When the phosphorus application rate reached 150 mg·kg^-1^, the excess phosphorus impaired the photosynthesis process of alfalfa plants, which in turn reduced the net photosynthetic rate of alfalfa (Song et al., 2021). Therefore, reasonable fertilization strategies can help improve plant photosynthesis.

The results showed that when the amount of double fungi and phosphorus application was 100 mg·kg^-1^, the light use efficiency and the instantaneous water use efficiency of leaves of alfalfa increased at the lower dose of P (P_0_-P_2_), while it decreased at the higher dose of P (P_2_-P_3_), and the net photosynthetic rate of leaves was significantly positively correlated with the light use efficiency and instantaneous water use efficiency (*p* < 0.05), which may be because phosphorus application and double fungus can improve the drought resistance of alfalfa. The lower the decrease of net photosynthetic rate, the less damage to photosynthetic organs in plant leaves, the higher the activity of photosynthetic cells, and the stronger the drought resistance of plants. Water use efficiency is an important symbol to judge the drought resistance of plants, which can reflect the water consumption and water use ability of plants. The higher the water use efficiency is, the stronger the adaptability of plants to drought is (Tang et al., 2023). Studies have shown that plants reduce chlorophyll content and stomatal conductance during drought stress, which affects plant photosynthetic capacity, which in turn reduces biomass (Naylor et al., 2018). Therefore, the appropriate bacterial fertilizer strategy is helpful to improve the drought resistance of plants.

Chlorophyll content is a key factor affecting the photosynthetic efficiency of alfalfa, and participates in the process of absorption, transfer and conversion of light energy (Zhu et al., 2017). Therefore, the chlorophyll content of plants plays an important role in their growth. The results showed that AMF inoculation of *Robinia* spp. seedlings significantly improved leaf area, carboxylation efficiency, chlorophyll content, net photosynthetic rate and effective photochemical efficiency of PSII (Mo et al., 2016), thereby maximizing their biomass (Zhu et al., 2014). Arbuscular mycorrhizal fungi can modulate the source-library relationship by enhancing the exchange of carbohydrates and mineral nutrients with the plant host, thereby stimulating the plant host to increase the rate of photosynthesis and increasing the carbon demand of arbuscular mycorrhizal fungi (Zhao et al., 2022b). Therefore, inoculation of arbuscular mycorrhizal fungi helps to increase chlorophyll content, promote photosynthesis, and thus increase crop yield.

### 4.3 The membership function comprehensively evaluates the optimal phosphorus application mode of different treatment combinations

Determining the best bacterial phosphorus treatment for the agricultural environment will help to improve the economic benefits of alfalfa. The effects of inoculation of Fm and Ge on dry matter yield, chlorophyll content and photosynthesis of alfalfa were different, and the membership function analysis and evaluation method was to comprehensively evaluate the performance of different treatments on each index and screen out the optimal phosphorus sterilization treatment among all treatments (Sun et al., 2019). In this study, the optimal combination of different treatments was ordered by T_3_P_2_ treatment (**Table 2**), indicating that when the phosphorus application rate was 100 mg·kg^-1^, the photosynthetic efficiency and dry matter yield of mixed inoculation of *Funneliformis mosseae* and *Glomus etunicatum* were maximized. Simultaneous inoculation of two fungi under suitable phosphorus application conditions can effectively promote the uptake of available phosphorus in the soil by plants, and then increase the dry matter yield of alfalfa.

## 5 Conclusion

Compared with no fertilization, the amount of phosphorus (P_2_O_5_) applied was 100 mg·kg^-1^, and the mixed inoculation of *Funneliformis mosseae* and *Glomus etunicatum* could significantly improve the photosynthetic efficiency and chlorophyll content of alfalfa leaves, thereby increasing the dry matter yield of alfalfa. This study clarified the optimal coupling mode of bacterial phosphorus for further improvement of alfalfa yield. It is the focus of future research to combine the anatomical structure and photosynthetic performance of alfalfa leaves and stems, and to clarify the synergistic mechanism of the anatomical structure and photosynthetic performance of alfalfa.

## Data availability statement

The original contributions presented in the study are included in the article/supplementary material. Further inquiries can be directed to the corresponding authors.

## Author contributions

DX: Conceptualization, Software, Writing – original draft, Writing – review & editing. XA: Data curation, Investigation, Methodology, Software, Writing – review & editing. IL: Supervision, Writing – review & editing. CM: Conceptualization, Writing – review & editing. QZ: Formal Analysis, Funding acquisition, Project administration, Resources, Validation, Visualization, Writing – review & editing.

## References

[B1] AbdElgawad H. El-Sawah A. M. Mohammed A. E. Alotaibi M. O. Yehia R. S. Selim S.. (2022). Increasing atmospheric CO_2_ differentially supports arsenite stress mitigating impact of arbuscular mycorrhizal fungi in wheat and soybean plants. Chemosphere 296, 134044. doi: 10.1016/j.chemosphere.2022.134044 35202662

[B2] Alotaibi M. O. Saleh A. M. Sobrinho R. L. Sheteiwy M. S. El-Sawah A. M. Mohammed A. E.. (2021). Arbuscular mycorrhizae mitigate aluminum toxicity and regulate proline metabolism in plants grown in acidic soil. J. Fungi 7, 531. doi: 10.3390/jof7070531 PMC8304902 34209315

[B3] Balestrini R. Brunetti C. Chitarra W. Nerva L. (2020). Photosynthetic traits and nitrogen uptake in crops: which is the role of arbuscular mycorrhizal fungi? Plants 9, 1105. doi: 10.3390/plants9091105 32867243 PMC7570035

[B4] Chen R. Liu G. Zhong W. Sun W. Zhang L. Hu Z.. (2015). Effect of ammonium polyphosphate on plant growth development and absorption of phosphorus and zinc in corn seedlings. Agric. Sci. Technol. 16 (08), 1716–1719. doi: 10.16175/j.cnki.1009-4229.2015.08.033

[B5] Cui L. E. Yang H. (2011). Accumulation and residue of napropamide in alfalfa (*Medicago sativa*) and soil involved in toxic response. J. hazard. mater. 190, 81–86. doi: 10.1016/j.jhazmat.2011.02.086 21439724

[B6] Frosi G. Barros V. A. Oliveira M. T. Cavalcante U. M. T. Maia L. C. Santos M. G. (2016). Increase in biomass of two woody species from a seasonal dry tropical forest in association with AMF with different phosphorus levels. Appl. Soil Ecol. 102, 46–52. doi: 10.1016/j.apsoil.2016.02.009

[B7] Gao X. Shi D. Y. Lv A. Wang S. Y. Yuan S. L. Zhou P.. (2016). Increase phosphorus availability from the use of alfalfa (*Medicago sativa L*) green manure in rice (*Oryza sativa L.*) agroecosystem. Sci. Rep. 6, 36981. doi: 10.1038/srep36981 27833163 PMC5105083

[B8] Hinsinger P. (2001). Bioavailability of soil inorganic P in the rhizosphere as affected by root-induced chemical changes: a review. Plant Soil 237, 173–195. doi: 10.1023/A:1013351617532

[B9] Li S. Yang W. Guo J. Li X. Lin J. Zhu X. (2020). Changes in photosynthesis and respiratory metabolism of maize seedlings growing under low temperature stress may be regulated by arbuscular mycorrhizal fungi. Plant Physiol. Biochem. 154, 1–10. doi: 10.1016/j.plaphy.2020.05.025 32505784

[B10] Liu J. Liu X. Zhang Q. Li S. Ma C. (2020). Response of alfalfa growth to arbuscular mycorrhizal fungi and phosphate-solubilizing bacteria under different phosphorus application levels. AMB Express 10, 1–3. doi: 10.1186/s13568-020-01137-w PMC7609620 33141419

[B11] Liu X. S. Zhao J. Liu J. Lu W. Ma C. Zhang Q. (2021). Water-phosphorus coupling enhances fine root turnover and dry matter yield of alfalfa under drip irrigation. Agron. J. 113 (5), 4161–4175. doi: 10.1002/agj2.20782

[B12] Mickky B. M. Abbas M. A. El-Shhaby O. A. (2018). Alterations in photosynthetic capacity and morpho-histological features of leaf in alfalfa plants subjected to water deficit-stress in different soil types. Indian J. Plant Physiol. 23, 426–443. doi: 10.1007/s40502-018-0383-7

[B13] Mo Y. Wang Y. Yang R. Zheng J. Liu C. Li H.. (2016). Regulation of plant growth, photosynthesis, antioxidation and osmosis by an arbuscular mycorrhizal fungus in watermelon seedlings under well-watered and drought conditions. Front. Plant Sci. 7, 644. doi: 10.3389/fpls.2016.00644 27242845 PMC4862978

[B14] Nasar J. Khan W. Khan M. Z. Gitari H. I. Gbolayori J. F. Moussa A. A.. (2021). Photosynthetic activities and photosynthetic nitrogen use efficiency of maize crop under different planting patterns and nitrogen fertilization. J. Soil Sci. Plant Nutr. 21, 2274–2284. doi: 10.1007/s42729-021-00520-1

[B15] Naylor D. Coleman-Derr D. (2018). Drought stress and root-associated bacterial communities. Front. Plant Sci. 8, 2223. doi: 10.3389/fpls.2017.02223 PMC5767233 29375600

[B16] Ning L. H. Du W. K. Song H. N. Shao H. B. Qi W. C. Sheteiwy M. S. A.. (2019). Identification of responsive miRNAs involved in combination stresses of phosphate starvation and salt stress in soybean root. Environ. Exp. Bot. 167, 103823. doi: 10.1016/j.envexpbot.2019.103823

[B17] Niyungeko C. Liang X. Liu C. Liu Z. Sheteiwy M. Zhang H.. (2018). Effect of biogas slurry application rate on colloidal phosphorus leaching in paddy soil: A column study. Geoderma 325, 117–124. doi: 10.1016/j.geoderma.2018.03.036

[B18] Qi M. Liu X. Zhang X. Liu Y. (2013). Effects of different phosphorus levels on photosynthesis and root nodule nitrogen-fixing characteristic of alfalfa. Acta Agrestia Sin. 21, 512. doi: 10.11733/j.issn.1007-0435.2013.03.016

[B19] Qin Y. Zhang W. Feng Z. Feng G. Zhu H. Yao Q. (2022). Arbuscular mycorrhizal fungus differentially regulates P mobilizing bacterial community and abundance in rhizosphere and hyphosphere. Appl. Soil Ecol. 170, 104294. doi: 10.1016/j.apsoil.2021.104294

[B20] Shan L. W. Zhang Q. Zhu R. F. Knog X. L. Chen J. S. (2020). Effects of AMF on growth and photosynthetic physiological characteristics of *Leymus chinensis* and *Medicago sativa* with and without nitrogen and phosphorus application. Acta Pratacult. Sin. 29, 46. doi: 10.11686/cyxb2019459

[B21] Sheteiwy M. S. El-Sawah A. M. Korany S. M. Alsherif E. A. Mowafy A. M. Chen J.. (2022). Arbuscular Mycorrhizal Fungus “*Rhizophagus irregularis*” impacts on physiological and biochemical responses of ryegrass and chickpea plants under beryllium stress. Environ. pollut. 315, 120356. doi: 10.1016/j.envpol.2022.120356 36220578

[B22] Song Y. li G. Lowrie R. (2021). Leaf nitrogen and phosphorus resorption improves wheat grain yield in rotation with legume crops in south-eastern Australia. Soil Tillage Res. 209, 104978. doi: 10.1016/j.still.2021.104978

[B23] Sun B. Gao Y. Wu X. Ma H. Zheng C. Wang X.. (2020). The relative contributions of pH, organic anions, and phosphatase to rhizosphere soil phosphorus mobilization and crop phosphorus uptake in maize/alfalfa polyculture. Plant Soil. 447, 117–133. doi: 10.1007/s11104-019-04110-0

[B24] Sun Y. L. Wei K. Q. Liu X. S. Zhao J. W. Li S. Y. Ma C. H.. (2022). Diurnal changes in photosynthesis and photosynthetic product partitioning in alfalfa in response to phosphorus application. Acta Pratacult. Sin. 31 (12), 85. doi: 10.11686/cyxb2021489

[B25] Sun Y. Zhang Q. Miao X. Liu J. Yu L. Ma C. (2019). Effects of phosphorus-solubilizing bacteria and arbuscular mycorrhizal fungi on production performance and root biomass of alfalfa. Sci. Agricult. Sin. 52 (13), 2230–2242. doi: 10.3864/j.issn.0578-1752.2019.13.004

[B26] Tang D. An Y. Cheng P. Li H. Yang J. Wang K. (2023). Responses of photosynthetic characteristics of typical shrubs in piedmont on the northern slope of tianshan mountains to drought stress. Xinjiang Agricult. Sci. 60 (6), 1531. doi: 10.6048/j.issn.1001-4330.2023.06.028

[B27] Valkov V. T. Sol S. Rogato A. Chiurazzi M. (2020). The functional characterization of LjNRT2.4 indicates a novel, positive role of nitrate for an efficient nodule N_2_-fixation activity. New Phytol. 228 (2), 682–696. doi: 10.1111/nph.16728 32542646

[B28] Yang H. Fang C. Li Y. Wu Y. Fransson Y. P. Rillig M. C.. (2022). Temporal complementarity between roots and mycorrhizal fungi drives wheat nitrogen use efficiency. New Phytol. 236, 1168–1181. doi: 10.1111/nph.18419 35927946

[B29] Yooyongwech S. Samphumphuang T. Theerawitaya C. Cha-Um S. (2014). Physio-morphological responses of sweet potato [*Ipomoea batatas* (L.) Lam.] genotypes to water-deficit stress. Plant Omics 7, 361–368. doi: 10.3316/informit.728000003198078

[B30] Yooyongwech S. Samphumphuang T. Tisarum R. Theerawitaya C. Cha-Um S. (2016). Arbuscular mycorrhizal fungi (AMF) improved water deficit tolerance in two different sweet potato genotypes involves osmotic adjustments *via* soluble sugar and free proline. Sci. Hortic. 198, 107–117. doi: 10.1016/j.scienta.2015.11.002

[B31] Zhang F. He H. Yu L. Lu W. Zhang Q. Ma C. (2017a). Nutritional quality of four important herbage species in summer grazing grassland in the alpine zone, west Tianshan Mountain. Acta Pratacult. Sin. 26 (8), 207–215. doi: 10.11686/cyxb2016389

[B32] Zhang Y. Wang J. Gong S. Xu D. Sui J. (2017b). Nitrogen fertigation effect on photosynthesis, grain yield and water use efficiency of winter wheat. Agric. Water Manage. 179, 277–287. doi: 10.1016/j.agwat.2016.08.007

[B33] Zhang L. Xu M. Liu Y. Zhang F. Hodge A. Feng G. (2016). Carbon and phosphorus exchange may enable cooperation between an arbuscular mycorrhizal fungus and a phosphate-solubilizing bacterium. New Phytol. 210 (3), 1022–1032. doi: 10.1111/nph.13838 27074400

[B34] Zhao J. Huang R. Yang K. Ma C. Zhang Q. (2022a). Effects of nitrogen and phosphorus fertilization on photosynthetic properties of leaves and agronomic characters of alfalfa over three consecutive years. Agriculture 12 (8), 1187. doi: 10.3390/agriculture12081187

[B35] Zhao J. W. Li S.Y. Sun Y. L. Liu X. S. Ma C.H. Zhang Q. B. (2022). Fine root turnover of alfalfa in different soil horizons under different nitrogen and phosphorus levels. Acta Prataculturae Sin. 31 (9), 118–128. doi: 10.11686/cyxb2021340

[B36] Zhu X. Q. Tang M. Zhang H. Q. (2017). Arbuscular mycorrhizal fungi enhanced the growth, photosynthesis, and calorific value of black locust under salt stress. Photosynthetica 55 (2), 378–385. doi: 10.1007/s11099-017-0662-y

[B37] Zhu X. Q. Wang C. Y. Chen H. Tang M. (2014). Effects of arbuscular mycorrhizal fungi on photosynthesis, carbon content, and calorific value of black locust seedlings. Photosynthetica 52, 247–252. doi: 10.1007/s11099-014-0031-z

